# Antiproliferative Effects of Oxytocin and Desmopressin on Canine Mammary Cancer Cells

**DOI:** 10.3389/fvets.2016.00119

**Published:** 2016-12-26

**Authors:** Micaela Andrea Benavente, Carolina Paula Bianchi, Fernanda Imperiale, Marcelo Alfredo Aba

**Affiliations:** ^1^Laboratorio de Endocrinología, Centro de Investigación Veterinaria de Tandil (CIVETAN), CONICET, Facultad de Ciencias Veterinarias, Universidad Nacional del Centro de la Provincia de Buenos Aires (UNCPBA), Tandil, Argentina; ^2^Laboratorio de Farmacología, Centro de Investigación Veterinaria de Tandil (CIVETAN), CONICET, Facultad de Ciencias Veterinarias, Universidad Nacional del Centro de la Provincia de Buenos Aires (UNCPBA), Tandil, Argentina

**Keywords:** mammary gland tumors, canines, cell line, oxytocin, desmopressin

## Abstract

Neoplasms of the mammary gland represent the most frequent tumor type in the female dog, and according to the histologic criteria, approximately 50% of them are malignant. In the most aggressive cases of mammary cancer, surgery is not enough to warrant a favorable outcome, and adjuvant therapies are needed to improve the patient’s overall survival. The aim of the present study was to evaluate the effects of two peptides on proliferation of a canine mammary cancer cell line derived from a simple carcinoma. The cell line CMT-U27 was grown in 96-well plates, at two cell densities (4 × 10^3^ and 8 × 10^3^ cells/well). Cultures were treated with oxytocin (OT) or desmopressin at five concentrations (10, 50, 100, 500, and 1000 nM). After 72 h of incubation, cell proliferation was determined by the MTT assay. Results showed that with 4 × 10^3^ cells/well, OT at 50, 500, and 1000 nM was growth inhibitory for the cells, being statistically significant at 1000 nM. On the contrary, no antiproliferative effect was observed with 10 or 100 nM. At 8 × 10^3^ cells/well, OT showed a significant antiproliferative effect only with the highest concentration (1000 nM). Desmopressin at 4 × 10^3^ cells/well decreased cell viability at concentrations of 50, 100, 500, and 1000 nM (statistically significant with the highest concentration), while no effect was observed with 10 nM. With 8 × 10^3^ cells/well, this peptide reduced cell growth at 100, 500, and 1000 nM. In conclusion, we suggest that these peptides may be potential and promising compounds for the treatment of dogs with simple carcinomas of the mammary gland. *In vivo* studies are required to confirm this hypothesis.

## Introduction

Mammary gland tumors are the most common neoplasms in intact female dogs, and approximately 50% of them are malignant. The treatment of choice for mammary gland tumors is surgical excision. However, in malignant cases, surgery alone is not enough to warrant a favorable outcome, and adjuvant therapies are needed to reduce the risk of recurrence or metastasis (1, 2). In women with aggressive breast cancer, postoperative chemotherapy is routinely administered to improve disease free and overall survival rates (3). In bitches, although several chemotherapy protocols have been tested (4–6), most of them did not show real benefits in survival time or have high toxicity and side effects. A recent study has described a longer overall survival in dogs treated with surgery and carboplatin, than those treated with surgery alone, indicating that this chemotherapeutic agent may be beneficial for the treatment of malignant mammary tumors (7).

Hormonal therapy is a well-established adjuvant treatment for women with estrogen receptor—positive tumors (hormone-dependent breast cancer), and tamoxifen is one of the antiestrogens most commonly used (8). However, the use of tamoxifen is restricted in female dogs due to its side effects, which includes vulvar swelling, vulvar discharge, and pyometra, among others (9). Therefore, it is essential to concentrate on the development of safe and effective adjuvant treatments for dogs with mammary cancer, in order to reduce the risk of recurrence or metastasis.

A number of *in vitro* studies performed on murine and human cancer cells have suggested that some peptide hormones can modulate tumor growth (10–12), by interacting with its membrane receptors. However, little is known about the effects of these peptides on canine mammary cancer.

Oxytocin (OT) is a peptide hormone mainly synthetized in the hypothalamus, which plays a key role in uterine contraction and milk ejection, among other functions (13). However, in recent years, a new role for OT has been described in relation to the carcinogenic process. Several studies have demonstrated that OT could stimulate, inhibit, or have no effect on neoplastic cell growth, and these diverse effects seem to be mediated by different signaling pathways. In neoplastic cells derived from trophoblast and endothelium, OT was found to promote cell proliferation (14, 15). On the contrary, in human neoplastic cells of mammary (16), nervous (17), and bone origin (18), OT inhibited the cell growth. Moreover, *in vivo* experiments showed that the subcutaneous administration of OT in Balb-c mice bearing breast carcinomas can reduce tumor growth (19). In addition, the presence of OT receptors has been described, both in human primary breast carcinomas and cell lines (20). According to these findings, OT appears to be involved in mammary tumor growth. To our knowledge, there is only one report about the effects of OT on the proliferation of canine mammary cancer cells.

Desmopressin [1-d-amino-8-d-arginine vasopressin (DDAVP)] is a synthetic analog of the antidiuretic hormone vasopressin, which has been used in humans and dogs for the management of diabetes insipidus (21). In addition, DDAVP has various effects in the hemostatic system, causing the release of coagulation factors VIII and von Willebrand (22). Due to its hemostatic properties, DDAVP has also been used in patients with different bleeding disorders (23). Interestingly, in a mouse model of breast cancer, DDAVP inhibited lung colonization by neoplastic cells (24). Since then, a number of studies have shown that this peptide seems to have antimetastatic and antiproliferative effects, both in mouse models of breast cancer and in different breast cancer cell lines (25, 26). Furthermore, a veterinary clinical trial has demonstrated that the perioperative administration of DDAVP increases the disease free and overall survival time in surgically treated bitches with mammary cancer of various histological types (27). Considering all these information, DDAVP could represent an excellent compound for surgical adjuvant therapy for the management of bitches with malignant mammary tumors.

The aim of the present study was to investigate the effects of OT and desmopressin on the proliferation of a canine mammary carcinoma cell line.

## Materials and Methods

### Tumor Cell Line

The canine mammary tumor cell line CMT-U27 was used in this study, which was generously supplied by Prof. Eva Hellmén. This cell line was established from a simple ductal carcinoma at the Swedish University of Agricultural Sciences (SLU) in Sweden (28). In previous studies, CMT-U27 cells have shown a high growth rate and antiapoptotic potential (29).

### Cell Culture Conditions

CMT-U27 cells were routinely cultivated in RPMI-1640 medium (Sigma-Aldrich) supplemented with 10% fetal bovine serum (FBS) (Natocor, Argentina), 100 UI/ml penicillin and 100 µg/ml streptomycin (Sigma-Aldrich). Cells were cultured in 25 cm^2^ cell culture plastic flasks (Corning Inc., USA) in a humidified incubator (Panasonic, Lobov Científica, Argentina) with 5% CO_2_ at 37°C.

### Peptides

Oxytocin was supplied by Chemo Romikin S.A. (Buenos Aires, Argentina) and desmopressin (Elea Laboratories, Argentina) was kindly provided by Dr. Daniel Alonso, from Laboratory of Molecular Oncology, Quilmes National University (Buenos Aires, Argentina). Both peptides were supplied in the lyophilized form, and at the beginning of the assays they were dissolved in sterile phosphate buffer saline (PBS) pH 7.4 at a final concentration of 100 µM, and then aliquots were stored at −20°C for no more than 1 month.

### Cell Proliferation Assay

Cell viability was measured using the MTT (3-(4,5-dimethylthiazol-2-yl)-2,5-diphenyltetrazolium bromide) colorimetric dye reduction method, as previously reported (26, 30). The medium was removed from the flasks, cells were washed with 3 ml of sterile PBS and then detached using EDTA for 5 min. Cell number was determined using a hemocytometer. Then, CMT-U27 cells were seeded into 96-well plates at two densities (4 × 10^3^ and 8 × 10^3^ cells/well) in 200 µl of RPMI-1640 supplemented with 10% (v/v) FBS and incubated in a humidified atmosphere at 37°C under 5% CO_2_ and 95% air to allow cell adhesion. After overnight culture, the medium was removed and replaced with 200 µl of medium containing OT or DDAVP at five concentrations: 10, 50, 100, 500, and 1000 nM, or medium without drugs (controls). The cells were incubated for 72 h. Afterward, MTT (Sigma-Aldrich) (20 µl) was added to each well and incubated for 2 h at 37°C to allow MTT to form formazan crystals by reacting with metabolically active cells. To complete solubilization of the formazan crystals, 100 µl of isopropanol were immediately added to each well. Cell viability was quantified by measuring the absorbance at 595 nm in a multi-well plate reader (Beckman Coulter, DTX 880 Multimode Detector). All the samples were examined in triplicate, and each experiment was conducted four times. The optical density of formazan formed in untreated control cells was considered as 100% viability. The optical densities from the treated cells were converted to a percentage of living cells against the control.

### Statistical Analyses

Data from the four independent experiments was analyzed by one-way analysis of variance contrasted with Dunnett Multiple Comparisons Test at a significance level of *p* < 0.05. The values were evaluated for approximate normality of distribution by the Kolmogorov–Smirnov test.

## Results

### Effect of OT on Canine Mammary Cancer Cells

**Figure 1:**
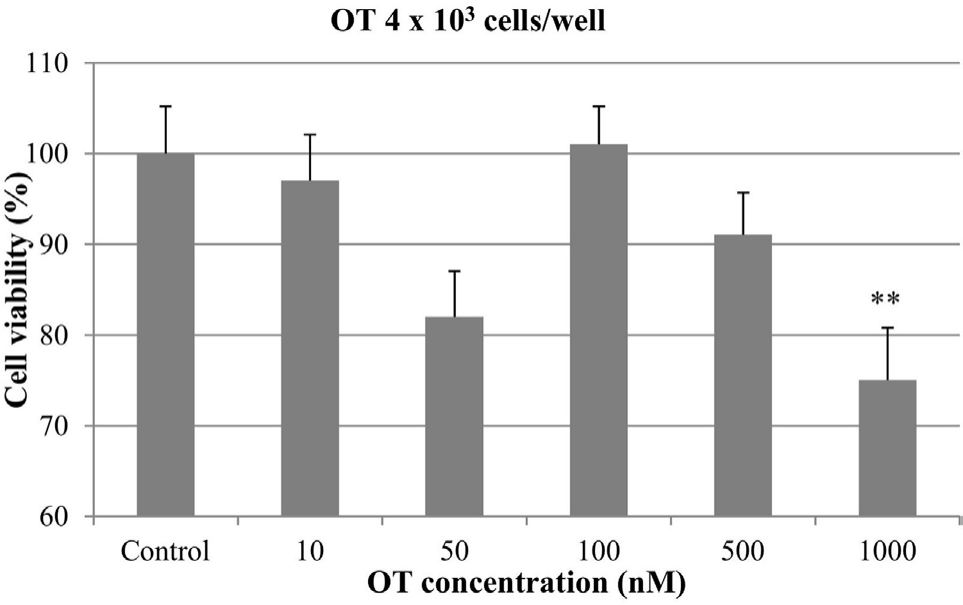
**Effect of oxytocin (OT) on *in vitro* growth of canine mammary cancer cells**. CMT-U27 cells were grown in 96-well plates (4 × 10^3^ cells/well) with appropriate concentrations of OT. MTT assay was performed after 72 h. (**) Indicates values statistically different from the control group (*p* < 0.01). Values represent means ± ΣE from 12 replicate measurements from four independent experiments.

At 4 × 10^3^ cells/well, a decrease in cell growth was observed with OT at concentrations of 50, 500, and 1000 nM. However, the inhibitory effect was statistically significant only with the highest concentration of the hormone (*p* < 0.01), which resulted in a 25% of inhibition of cell viability. OT did not affect the proliferation of CMT-U27 cells at 10 and 100 nM (Figure 1).

**Figure 2:**
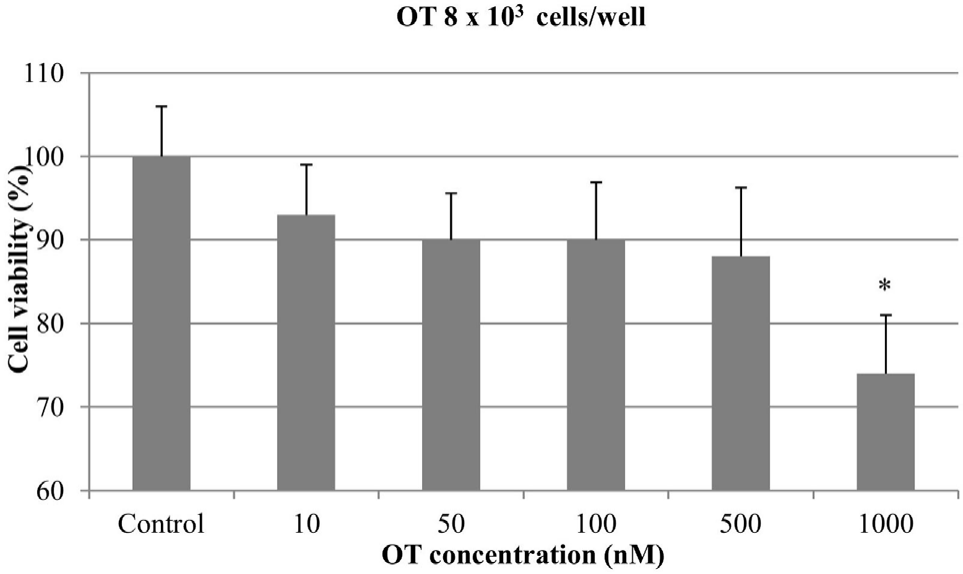
**Effect of oxytocin (OT) on *in vitro* growth of canine mammary cancer cells**. CMT-U27 cells were grown in 96-well plates (8 × 10^3^ cells/well) with appropriate concentrations of OT. MTT assay was performed after 72 h. (*) Indicates values statistically different from the control group (*p* < 0.05). Values represent means ± SE from 12 replicate measurements from four independent experiments.

Regarding the experiments with 8 × 10^3^ cells/well, no significant antiproliferative effect was obtained with OT at 10, 50, 100, and 500 nM. On the contrary, the highest concentration of OT (1000 nM) caused a significant inhibition of CMT-U27 cell growth (almost 25%) (*p* < 0.05) (Figure 2).

### Effect of Desmopressin on Canine Mammary Cancer Cells

**Figure 3:**
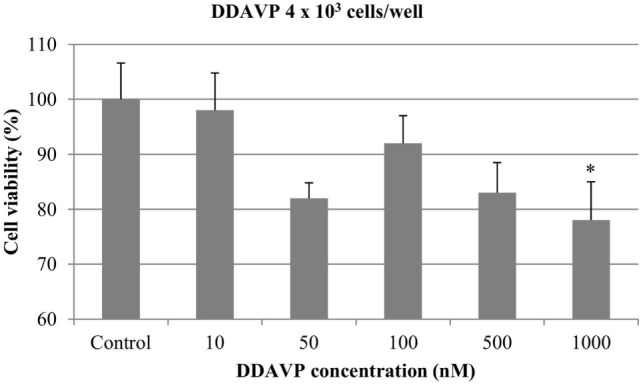
**Effect of desmopressin [1-d-amino-8-d-arginine vasopressin (DDAVP)] on *in vitro* growth of canine mammary cancer cells**. CMT-U27 cells were grown in 96-well plates (4 × 10^3^ cells/well) with appropriate concentrations of DDAVP. MTT assay was performed after 72 h. (*) Indicates values statistically different from the control group (*p* < 0.05). Values represent means ± SE from 12 replicate measurements from four independent experiments.

At 4 × 10^3^ cells/well, DDAVP at concentration of 1000 nM resulted in a significant antiproliferative effect (22%) (*p* < 0.05). The growth of mammary cancer cells was not affected by the addition of DDAVP at 10, 50, 100, and 500 nM (Figure 3).

**Figure 4:**
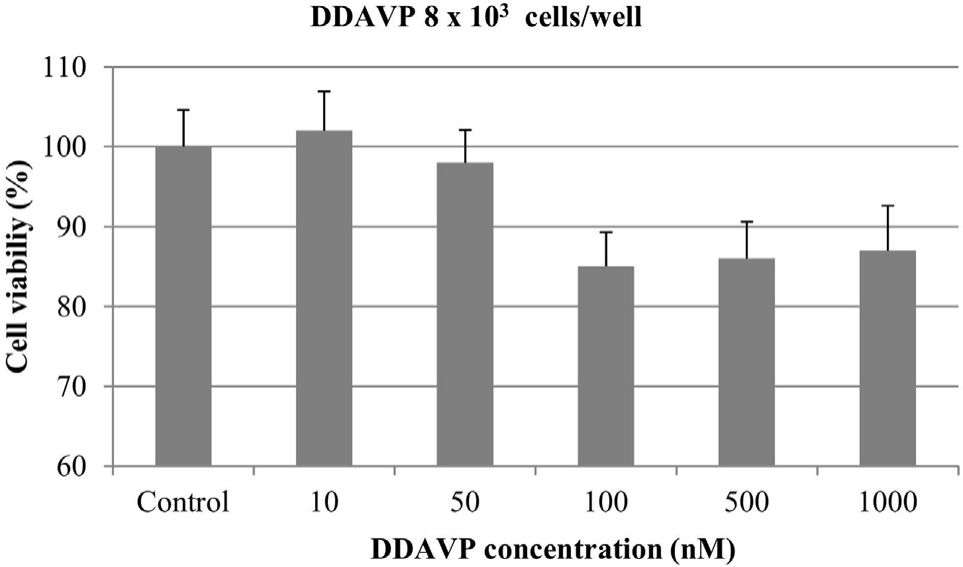
**Effect of desmopressin [1-d-amino-8-d-arginine vasopressin (DDAVP)] on *in vitro* growth of canine mammary cancer cells**. CMT-U27 cells were grown in 96-well plates (8 × 10^3^ cells/well) with appropriate concentrations of DDAVP. MTT assay was performed after 72 h. Values represent means ± SE from 12 replicate measurements from four independent experiments.

At 8 × 10^3^ cells/well, nearly 15% of cell growth inhibition was obtained with DDAVP at 100, 500, and 1000 nM. However, this difference was not statistically significant (*p* > 0.05). DDAVP at 10 and 50 nM did not show any significant effect on cell growth (Figure 4).

## Discussion

This study provides information about the effects of two peptides on *in vitro* growth of a canine mammary simple carcinoma cell line. The present results show that treatment of CMT-U27 cells with OT and DDAVP inhibited the proliferation of tumor cells.

As stated before, this canine cell line was isolated from a simple carcinoma, a histological subtype frequently diagnosed in female dogs, and associated with a short disease-free period after surgery (31). In previous studies, CMT-U27 cells showed to be sensitive to antiprogestins (32) and also to cyclooxygenase inhibitors and doxorubicin (33). For this reason, this cell line seems to be a useful *in vitro* model to evaluate the effects of potential anticancer drugs.

To our knowledge, only one study reports on the effects of OT on this canine mammary cancer cell line, where a decrease in cell proliferation was observed at 1000 nM concentration, although this result was not consistent in all the assays performed (34). The author attributes these variations to different aspects of the cell line, including the stage of the cell cycle in which the cells were at the beginning of each assay, the moment in which the cells were last reseeded before the assay, or when the cell culture medium was changed in relation to the assay. Besides, this last study reported inhibitory effect with OT 100 nM. In the present study, a growth inhibitory effect with OT at 1000 nM was detected in all the assays, while no significant effect was observed at 100 nM. Both studies on canines are in agreement with experiments on human breast cancer cell lines, who reported the inhibitory effect of OT, even with minimum doses of 1 nM of OT (35). A more recent study demonstrated that both normal myoepithelial cells and breast carcinomas cell lines can synthesize OT and that estrogens can modulate this synthesis (36). Additional studies on CMT-U27 cells could include the evaluation of OT synthesis by the canine cancer cells and the effects of estrogens or progesterone on OT action.

This is the first report where the effects of desmopressin are evaluated on cultures of canine mammary cancer cells. When the cells were treated with increasing concentrations of DDAVP, a cell growth inhibition was observed. Previously, a study demonstrated a beneficial effect of perioperative administration of DDAVP (administered at doses of 1 µg/kg, 30 min before and 24 h after surgery) on survival in bitches with mammary cancer, without causing side effects (27). The results obtained in the present study seem to support the hypothesis that DDAVP can inhibit the growth of residual malignant cells, thus limiting tumor progression. More recently, a phase II dose-escalation trial in women with breast cancer was performed, demonstrating that DDAVP, administered 30–60 min before and 24 h after surgical resection, reduced intraoperative bleeding and the number of circulating tumor cells. Moreover, it appeared to be safe and well tolerated at the highest dose level evaluated (2 µg/kg) (37).

The *in vitro* results of the present study are in agreement with those of other studies that have shown that the addition of DDAVP to mouse and human mammary cancer cells inhibits cell proliferation (38, 39). The antiproliferative action of DDAVP appears to be mediated trough the V_2_ vasopressin receptor, which involves the increase of intracellular cAMP concentrations (22). The presence of V_2_ vasopressin receptors has been demonstrated in the MCF-7 human breast cancer cell line (40). More recently, the novel [V^4^Q^5^] DDAVP analog has been synthesized and assayed in cultures of MCF-7, causing a significantly greater inhibition of cell proliferation than DDAVP, at concentrations of 500 nM or higher (26). Additional studies are necessary to investigate the presence of the V_2_ vasopressin receptor on canine mammary cancer cell lines and spontaneous mammary tumors and to evaluate the effects of the analog [V^4^Q^5^] dDAVP on *in vitro* growth of canine mammary cancer cells.

Besides, our experiments showed that at the lower cell density (4 × 10^3^ cells/well), both OT and DDAVP 50 nM exhibited an antiproliferative effect similar to that obtained with 1000 nM (approximately 20%). Likewise, a biphasic pattern, observed at low cell density, has been reported recently on human prostate cancer cell lines treated with desmopressin, where low doses (1 nM) as well as a higher dose (1 µM) have an antiproliferative effect (41). This “biphasic effect” of peptides was shown some decades ago with OT on human uterus and oviduct (42). Afterward, this effect was described for other molecules in detail in 1977 (43). This biphasic dose response seems to occur when a single agonist has differential affinities for two opposite receptor subtypes, leading to different signaling pathways. Due to the fact that only one OT receptor is known, some authors have suggested that in some tissues and under particular circumstances, OT action could be mediated by vasopressin receptors, for which OT maintains a certain affinity (44).

To conclude, the present study shows that OT and desmopressin inhibit the proliferation of the canine mammary cancer cell line CMT-U27, by almost 20% with the highest concentration (1000 nM). Further studies on this and other canine mammary cell lines are needed to evaluate its potential as antitumor agents, alone or combined with conventional cytotoxic drugs, in the treatment of dogs with advanced mammary carcinomas.

## Author Contributions

MB: study design, conducted laboratory work, data collection and analysis, and drafting the manuscript. CB: study design, data analysis, and critical review of the manuscript. FI: conducted laboratory work and critical review of the manuscript. MA: study design and coordination, data analysis, drafting, and review of the manuscript.

## Conflict of Interest Statement

The authors declare that the research was conducted in the absence of any commercial or financial relationships that could be construed as a potential conflict of interest.
